# Efficacy of parent-infant psychotherapy compared to care as usual in children with regulatory disorders in clinical and outpatient settings: study protocol of a randomised controlled trial as part of the SKKIPPI project

**DOI:** 10.1186/s12888-021-03112-6

**Published:** 2021-02-27

**Authors:** Mona Katharina Sprengeler, Janna Mattheß, Melanie Eckert, Katharina Richter, Gabriele Koch, Thomas Reinhold, Petra Vienhues, Anne Berghöfer, Julia Fricke, Stephanie Roll, Thomas Keil, Christiane Ludwig-Körner, Lars Kuchinke, Kai von Klitzing, Franziska Schlensog-Schuster

**Affiliations:** 1 Department of Child and Adolescent Psychiatry, Psychotherapy and Psychosomatics, University of Leipzig, Liebigstrasse 20a, 04103 Leipzig, Germany; 2 International Psychoanalytic University Berlin, Berlin, Germany; 3 Institute of Social Medicine, Epidemiology, and Health Economics, Charité – Universitätsmedizin Berlin, Corporate Member of Freie Universität Berlin and Humboldt-Universität zu Berlin, Berlin, Germany; 4 Fachklinik für Psychiatrie, Psychosomatik und Psychotherapie, DIAKO Nordfriesland, Flensburg, Germany; 5 Institute for Clinical Epidemiology and Biometry, University of Würzburg, Würzburg, Germany; 6 State Institute of Health, Bavarian Health and Food Safety Authority, Bad Kissingen, Germany; 7 Psychological Methods and Evaluation, International Psychoanalytic University Berlin, Berlin, Germany

**Keywords:** Mental health, Clinical assessment, Focus-based psychodynamic intervention, Manualised short-term psychotherapy, Early childhood, Maternal sensitivity, Infant psychopathology

## Abstract

**Background:**

The first years of life are a significant period for child development, when children are particularly sensitive and prone to crises. This early phase lays the foundation for healthy growth. Clinical assessment of psychological symptoms in early infancy and adequate treatment are both important in improving the diagnostic outcome and preventing later long-term developmental consequences. The most common psychological problems in the first 3 years of life are regulatory disorders. The aim of this trial is to investigate the efficacy of Parent-Infant Psychotherapy (PIP) for infants and young children (aged 0–36 months, diagnosed with at least one regulatory disorder) and their mothers, compared to care as usual (CAU).

**Methods:**

In this open multicentre randomised controlled trial, 160 mother-infant dyads are randomised to receive PIP or CAU for 6 weeks of intervention in clinical or outpatient (including home treatment) settings. The primary outcome is the maternal sensitivity (sensitivity scale of the Emotional Availability Scales (EAS)) after 6 weeks. Secondary outcomes include assessment of interaction, mental health problems, attachment, development, psychological factors, treatment adherence, health care system utilisation, and costs, after 6 weeks and 12 months.

**Discussion:**

This study will evaluate whether a manualised focus-based short-term psychodynamic psychotherapeutic intervention in mother-child dyads improves the care situation for families of children diagnosed with regulatory disorders, and helps prevent long-term psychopathologies. Assessment of the intervention in different settings will support the development of more tailored interventions for affected infants and their mothers.

**Trial registration:**

German Clinical Trial Register, ID: DRKS00017008. Registered 03/20/2019.

## Background

The most common psychopathologies in early childhood are regulatory disorders (e.g. excessive crying, sleeping and feeding disorders), which affect about 20% of all infants and toddlers [1]. Fortunately, the majority of these early mental health problems are transient. Yet there are major gaps in improving clinical outcome and development, in (1) adequate assessments of early psychopathological signs [2, 3] due to a lack of validated assessment methods, and (2) detection of severe clinical courses and subsequent treatment [1, 4]. The distribution of psychopathology seems to correspond with later developmental stages, but the description of symptoms in the early years of life do not fit within the framework of the common diagnostic classification systems of ICD-10 and DSM-V. Only in the last couple of years has the focus of research in this field shifted to diagnostic criteria for infants, with the development of the Diagnostic Classification of Mental Health and Developmental Disorders of Infancy and Early Childhood DC: 0–5™ [5].

A meta-analysis by Hemmi and colleagues [1] showed that parental mental health problems (e.g. depression, anxiety, obsessive-compulsive disorders) are potential risk factors for infant psychopathology. Moreover, Fraiberg, Adelson and Shapiro [6] underline the problem of transmission in parenthood in their concept of the ‘Ghosts in the nursery’. They explain the negative impact on parental functions using their own transgenerational experience when raising their children [6, 7]. The development of a strong, stable bond between mother and child is one of the most essential factors for an infant’s resilience and healthy development [8, 9]. Maternal sensitivity is well known to be a key factor in the mother-infant interaction and the development of a secure attachment to the mother [8–10].

Psychodynamic Parent-Infant Psychotherapy (PIP) within dyadic or triadic sessions aims to improve maternal sensitivity and attachment security [11]. Various studies have already demonstrated the efficacy of PIP in (1) reducing psychological symptoms (e.g. [12]), (2) improving maternal sensitivity (EAS), and (3) improving attachment security in high-risk samples [11, 13] compared with control groups and diverse interventions (e.g. education, support and medication) [11, 14, 15].

Nevertheless, the authors note some limitations such as overall low study quality and heterogeneous samples without clinical marker cut-offs. The meta-analysis by Hemmi et al. [1] also criticises the fact that most of the studies on regulatory disorders have focused only on singular regulatory disorders without recording data for any other coexisting mental health problems.

Thus, despite the clear benefits of attachment- and sensitivity-related interventions for the mother-child dyad [16], a corresponding study of PIP with validation in inpatient psychiatric departments is still needed. Moreover, recent developments in child psychiatry provide evidence for the efficacy of home treatment or home-based interventions by psychotherapists [17–19] . Home-based PIP interventions have been demonstrated to be effective in improving children’s attachment organisation [20], but other randomised controlled trials (RCTs) have reported no effects on attachment measures or maternal sensitivity [21, 22]. It should clearly be noted that there are wide methodological differences between these studies.

In summary, previous RCTs on the efficacy of PIP have dealt exclusively with specific risk groups and/or have taken place in outpatient – but not yet in inpatient – settings [12, 14, 22–24]. These studies, using validated assessment methods, found an improvement in attachment security and long-term effects in a child’s development, as well as symptom reduction [15]. An exploration and validation of PIP in inpatient psychiatry and non-inpatient treatment (e.g. psychiatric outpatient clinics, home treatment) is still pending [25].

The aim of this study is to assess the efficacy of PIP in young children (0–36 months, diagnosed with at least one regulatory disorder) and their mothers in clinical and outpatient settings compared to care as usual (CAU). CAU includes different approaches, from regular therapy sessions to psychiatric and paediatric health care.

## Methods and design

### Overview

The trial is part of the SKKIPPI research project, which is designed to evaluate integrated psychological-psychiatric care for parents and their children in the first 3 years of life. The overall project consists of a cohort study and two randomised controlled trials (RCTs) [26], performed under the auspices of the German Health Care Innovation Fund, which aims to improve the quality and integration of health care [27]. The cohort study investigates the occurrence of mental health disorders, psychosocial problems and health care use in the first 3 years of life, taking random samples from registry offices of three German regions [28]. Of the two RCTs, one focuses on mothers with postpartum mental disorders, and their children [29], while the present study focuses on children with regulatory disorders, and their mothers.

### Aims and hypotheses

This trial evaluates the efficacy of PIP in comparison to CAU regarding maternal sensitivity. The intervention will be assessed in inpatient (Psychosomatics / Parent-infant unit; PIU) or outpatient psychiatric departments via home visits by trained and certified PIP psychotherapists. It is expected that CAU will reduce symptoms of mental health problems in mother and child, but have little or no effect on maternal sensitivity. In comparison to CAU, PIP is particularly expected to enhance maternal sensitivity following the intervention and to increase the rate of securely attached children at 12 months follow-up. Additional analyses will address health care economics and the use of German health care services by mothers with children who have regulatory disorders, and the groups will be compared.

### Trial design

The two-armed, open, randomised, controlled multicentre study with parallel groups and blinded endpoint assessment (PROBE design; prospective randomised, blinded endpoint) with a six-week intervention is currently underway in Leipzig, Berlin, Potsdam and Hamburg. All study centres and participating clinics there provide focused and supervised certified PIP in accordance with a previously elaborated SKKIPPI Study Manual (Schlensog-Schuster F, Koch G, Ludwig-Körner C: SKKIPPI Study manual for Parent-Infant-Toddler Psychotherapy (PIP), Unpublished). Depending on the severity of the child’s pathological symptoms, and in agreement with parental preferences, a decision is made on treatment setting (in−/outpatient) before randomisation. Participants are then randomly assigned to either PIP or CAU, and data are collected at three measurement points: at baseline before intervention, immediately after treatment, and at 12 months follow-up. The biostatistician of the Institute for Social Medicine, Epidemiology, and Health Economics at the Charité – Universitätsmedizin Berlin generates the allocation sequence based on a computer generated random list, applying a 1:1 block randomisation with variable block length and stratified by setting (inpatient/outpatient) and study centre. Mother-child dyads are randomly assigned to either PIP or CAU.

**Fig. 1:**
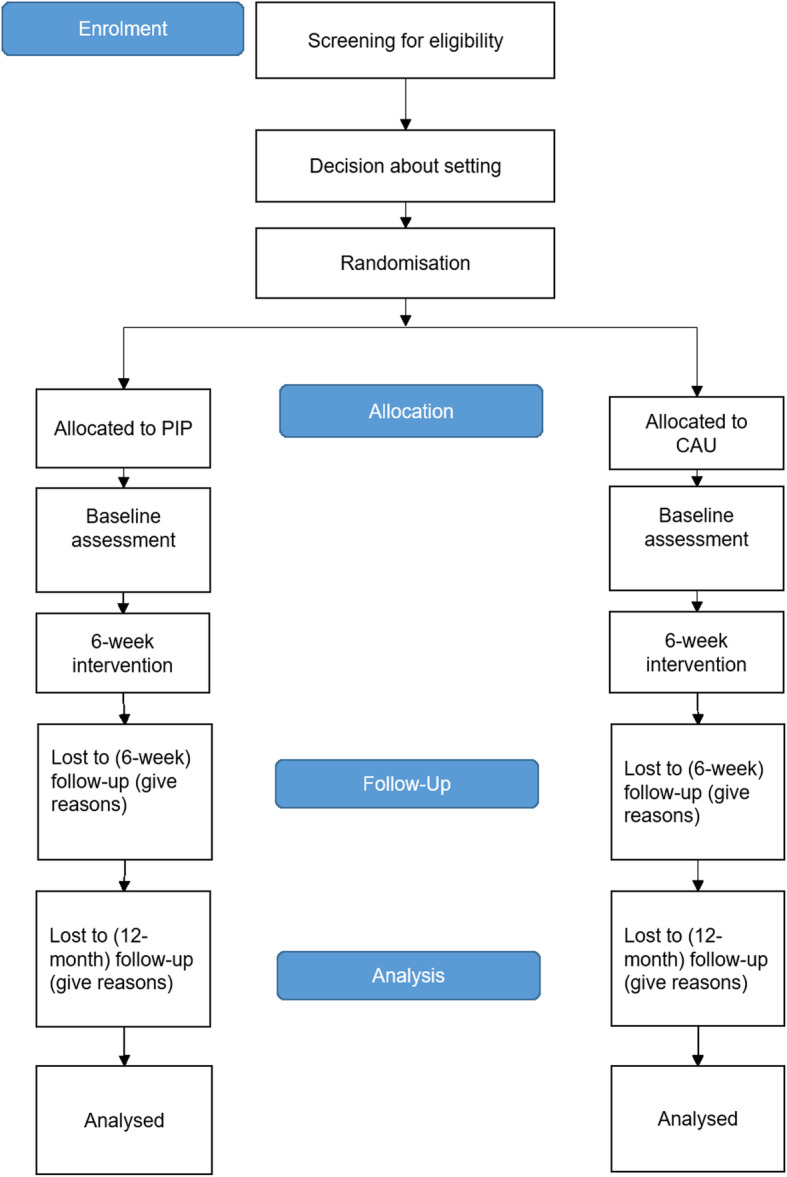
Standards of reporting trials (CONSORT). Diagram showing the planned flow of participants through the study

Before being enrolled in the trial, participants are screened by a professional physician or psychotherapist for eligibility and clinical diagnoses. Only children receiving an ICD-10 diagnosis of regulatory disorder can take part in the study. Eligible mothers of these children are informed verbally about the study, and written informed consent must be given before randomisation. Afterwards, baseline assessments are carried out, and the 6 weeks of interventions start. At the end of treatment, participants are assessed again, as well as 12 months after baseline. Mothers will receive €100 as monetary compensation (25€ after Baseline, 25€ after 6 weeks follow-up and 50€ after the last assessment) until the 12-months follow-up. Throughout the study, the study nurse is in contact with the participants via phone calls or e-mails to schedule appointments. Treatment allocation is masked to the assigning personnel and there are no special criteria for discontinuing or modifying the allocated intervention (Fig. 1).

### Participants and eligibility screening

We include infants and young children aged 36 months or younger (Table 1). Participation is conditional upon written informed consent from the legal guardians. All participants are carefully informed about the study goals and assessment.

**Table 1:** Inclusion and exclusion criteria

Inclusion criteria	
• Infants aged 36 months or younger	
• Infants diagnosed (by a certified paediatrician, psychiatrist or psychotherapist) with at least one regulatory disorder	
• Mothers must speak German to a sufficient level and understand the aims of the study	
• Mothers must be in a psychological state that allows them to participate in the study	
Exclusion criteria	
• Mother or child is already participating in other clinical trials	
• Mother or child is receiving any other form of psychotherapy	
• Mother is dealing with acute substance abuse, or has suicidal tendencies or acute psychosis	
• Child has a suspected alcohol embryopathy, or severe organic impairments	

### Interventions

#### Parent-infant psychotherapy

Parent-Infant Psychotherapy (PIP) is a manualised focus-based short-term psychodynamic psychotherapeutic intervention that we have developed (Schlensog-Schuster F, Koch G, Ludwig-Körner C: SKKIPPI Study manual for Parent-Infant-Toddler Psychotherapy (PIP), Unpublished), which includes the children and their primary caregivers ([11, 25]; Schlensog-Schuster F, Koch G, Ludwig-Körner C: SKKIPPI Study manual for Parent-Infant-Toddler Psychotherapy (PIP), Unpublished). Generally, the therapist works with the parents (or other important caregivers) and the infant in joint sessions. In special situations (for example, if the mother has depressive symptoms) there may be a few sessions with one parent alone, in order to address important themes that cannot be addressed in joint sessions. The intervention focuses mainly on the meaning the parents ascribe to their child’s symptoms. The therapist observes the parent-child interaction and tries to link his/her observation to the parental beliefs and interpretations. The therapeutic focus includes the family history, parental thoughts, symptoms, and the therapeutic relationship that develops during the sessions. The aim of the therapist’s intervention is to help the parents to understand and to ‘contain’ the child’s affective states. The intervention builds on parental self-reflection and supports the parental ability to mentalise. PIP also aims to strengthen specific parental ego functions.

The focused psychodynamic psychotherapy is conducted in 12 sessions, twice a week over a period of 6 weeks. The course of PIP is divided into three phases: the initial diagnostic phase, the second phase of the focused intervention, and the third and final phase of integration. The therapists are trained to consider the parents’ couple relationship, the personalities of the parents, and possible conflicts in the mother-father-infant triads. The therapists try to create a holding environment for parents and infant within the therapeutic sessions. The holding therapeutic function helps participants to clarify problems, regulate affects, engage in self-reflection, and change their perspectives, and thus provides support for interpersonal resources in the parent-child triad. Psycho-education and the use of video feedback are additional therapeutic options for the intervention, which is oriented towards the mother’s psychodynamic, structural level. Video feedback can be used if there are maternal developmental deficits and a space of self-perception with the child cannot be created in inner presentations and phantasies.

**Fig. 2:**
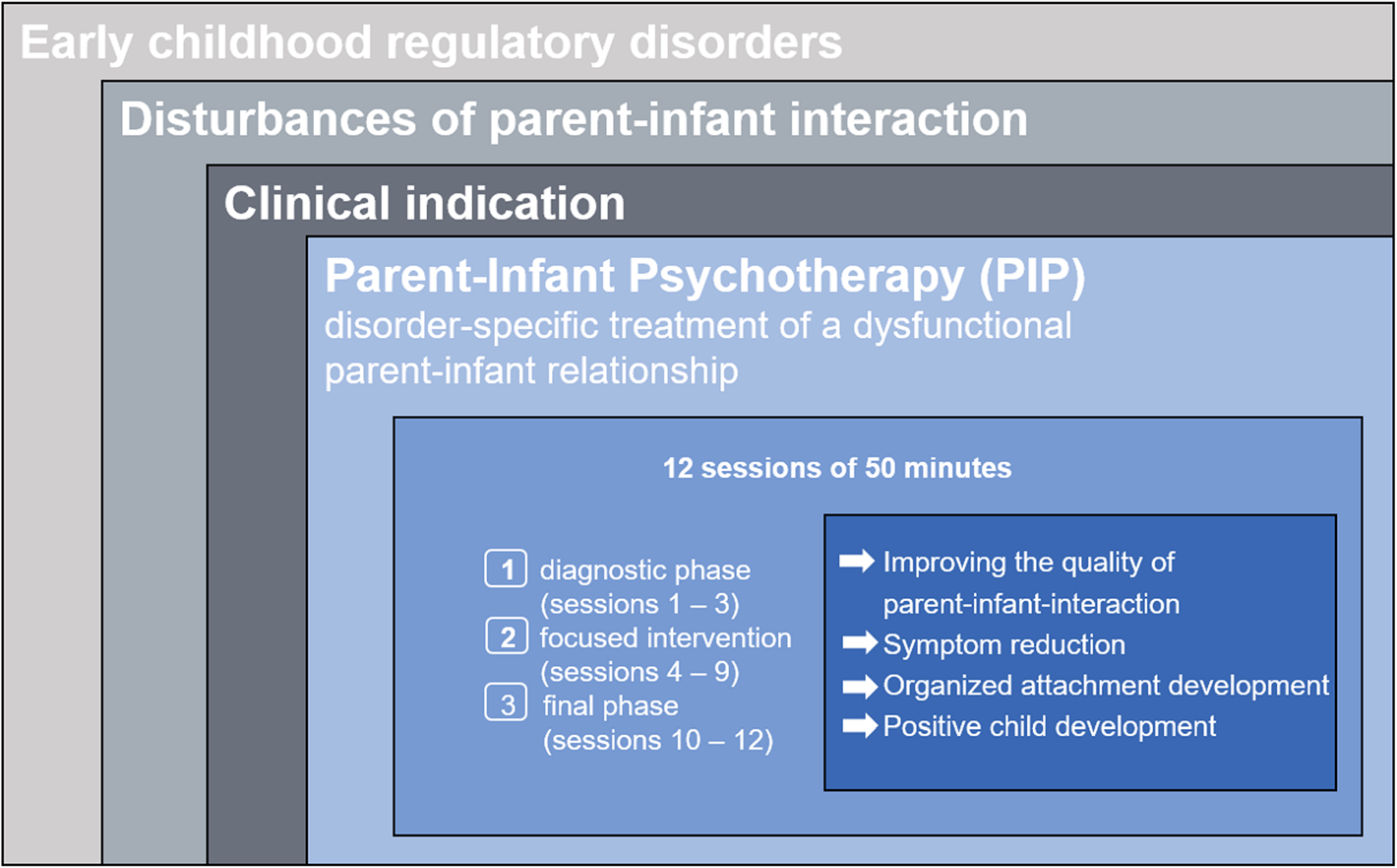
Indication and Procedure for Parent-Infant Psychotherapy

At the end of the intervention, the therapist encourages the parents to reflect upon their experience of the therapeutic process and to develop strategies for using the therapeutic experience in their everyday life. The treatment technique is manualised and follows guidelines according to the setting. Every therapist undergoes specialised training according to the manual before the first session to ensure adherence to the treatment techniques described within it. All therapists are either psychodynamic psychotherapists (psychologists and medical doctors) or psychologists and medical doctors in advanced psychodynamic psychotherapy training. The therapies are supervised by experienced PIP therapists at regular intervals in order to secure treatment fidelity (Fig. 2).

#### Control group: care as usual

Patients in the control group receive standard medical, pedagogical, and/or psychotherapeutic care for 6 weeks, as usually provided by the German health care system. Ethical considerations mean the control group could not be designed as a no treatment control. In the inpatient setting, more care might be provided than in outpatient settings. This could include counselling, physical or psychotherapy, and other psychiatric-therapeutic interventions. Outpatient settings may be either therapeutic or pedagogical care. Because of possible heterogeneities in the number and intensity of CAU treatments compared to PIP, all individual interventions will be documented in detail.

### Outcomes

#### Primary outcome

The primary outcome will be maternal sensitivity measured after 6 weeks of intervention. Videotaped dyadic sessions of free-play interaction will be coded using the subscale ‘sensitivity’ of the emotional availability scales (EAS) [30]. EAS assesses the quality of the mother-child interactions using a seven-point Likert scale for maternal sensitivity. Independent and reliable coders blind to treatment allocation rate all the 15- to 20-min video scenes.

### Secondary outcomes

#### Child attachment

The most important secondary outcome will be the child’s attachment style, measured using the Strange Situation Procedure (SSP) for children up to 20 months [31]. The SSP is a standardised procedure recording eight episodes to rate the child’s attachment quality to his or her caregiver. Attachment security is rated by independent coders blind to treatment allocation. The classification can be secure (B), avoidant (A), or ambivalent (C). Disorganised (D) attachment qualities will also be scored.

The attachment style of children older than 20 months is assessed using Attachment Q-Sort (AQS) [32]. The AQS evaluates the degree of attachment security for infants aged 12 to 48 months, and is a one- to two-hour procedure in which the child and its mother are observed in their home environment. The infant’s behaviour is evaluated by the observer for 90 standardised Q-Sort items, which are compared to a typical profile of securely attached children. The AQS varies on a scale between − 1 and 1, and does not reflect the variety of attachment categories, as the SSP does.

#### Child mental health problems

To quantify the child’s psychiatric symptoms, a multi-method approach is used, with reliable and valid questionnaires and a structured interview we have developed using the diagnostic criteria of the DC: 0–5 Axis 1 [5, 33]. DC: 0–5 interviews are conducted at 6 weeks follow-up, and 12 months follow-up. Mothers also complete the Child Behaviour Checklist (CBCL) [34] to detect the child’s emotional and behavioural problems. This 100-item screening instrument is validated at a child’s age of 18 months and re assessed at 6 weeks and 12 months follow-up. The child’s mental health symptoms are measured using the German questionnaire Crying, Feeding, Sleeping (SFS) (Gross S, Reck C, Thiel-Bonney C, Cierpka M: German version of the Questionnaire on Crying, Feeding, Sleeping, Unpublished). The SFS assesses problems in the child’s behavioural regulation and dysfunctional communication patterns, and the primary caregivers’ stress symptoms. The questionnaire is assessed at 6 weeks follow-up.

#### Maternal mental health problems

Maternal mental health problems are assessed using the Brief Symptom Checklist (BSCL) [35], a validated questionnaire to measure adult psychopathological symptoms, at 6 weeks follow-up. A further questionnaire assessing mental health status, the Anxiety Screening Questionnaire (ASQ-15) [36], covers all anxiety disorders diagnosed by DSM IV and ICD-10. Mothers complete the questionnaire at 6 weeks follow-up. The Scale for Impulsive Behaviour and Emotional Dysregulation of Borderline Personality Disorder (IES-27) [37] is a screening instrument to measure typical patterns of BPD. The IES-27 questionnaire is assessed at 6 weeks follow-up. Symptoms of maternal depression are measured using the Edinburgh Postnatal Depression Scale (EPDS) [38] at all measurement points. To explore psychiatric disorders, the Mini International Neuropsychiatric Interview (M.I.N.I.) [39], a structured diagnostic interview, which assesses 20 psychiatric disorders by DSM IV and ICD-10 diagnosis, is used. The M.I.N.I. assesses possible maternal clinical diagnoses and will be administered by trained interviewers at 12 months follow-up.

#### Maternal attachment and reflective functioning

Maternal attachment style and reflective functioning, i.e. the capacity to mentalise, are considered essential moderators in the present design and are assessed using the Adult Attachment Interview (AAI) [40]. The AAI is a semi-structured interview with precise psychometric properties to evaluate the maternal ‘state of mind’ with respect to attachment. The AAI is conducted by trained interviewers, transcribed, and subsequently scored by reliable and independent coders blind to treatment allocation.

#### Developmental status of the child

The standardised German developmental test ET 6–6 R [41] assesses the level of social-emotional, cognitive, motor and language development of children aged between 6 months and 6 years. The developmental status of the child is evaluated at all three measuring points. In addition, and at the Leipzig study centre subgroup only (due to availability reasons), the Bayley Scales of Infant and Toddler Development (Bayley-III) [42] is assessed. Bayley-III depicts the developmental level of infants aged between 0 and 42 months in a differentiated manner based on various subtests.

#### Psychological factors

The Parental Stress Index (PSI) [43] is used to measure maternal parental stress, and the Parental Reflective Functioning Questionnaire (PRFQ) [44] to measure the mother’s self-reported ability to mentalise in the context of her relationship to her child. Both questionnaires are assessed at 6 weeks, and 12 months follow-up.

Additionally, the five further subscales of the EAS based on the 15-min videotaped mother-child interactions (maternal dimensions: structuring, non-intrusiveness and non-hostility; and child dimensions: responsiveness to parent, involvement with parent) are analysed as secondary outcomes. Free-play sessions are recorded at all measurement time-points and rated by reliable and independent coders blind to treatment allocation.

#### Treatment adherence

Treatment Adherence is assessed using an in-house questionnaire with 30 items to evaluate the methods and interventions applied by the PIP therapist after each session.

#### Health care and social service utilisation

**Fig. 3:**
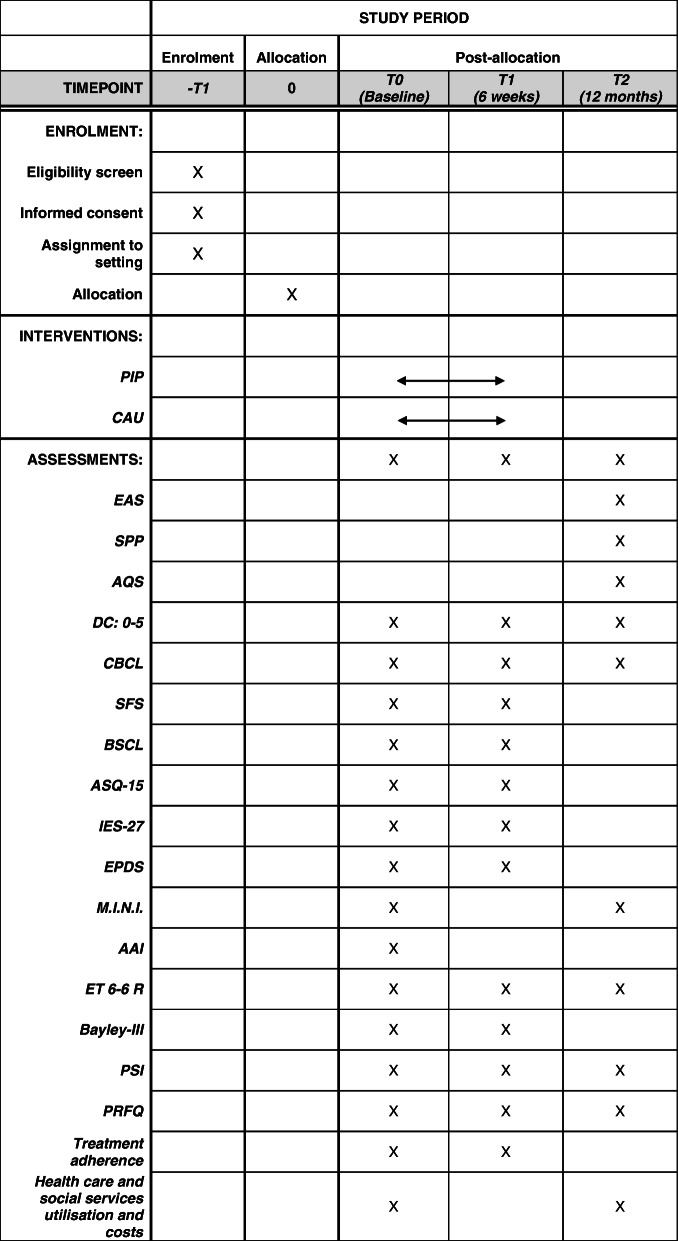
Full Standard Protocol Items: Recommendations for Interventional Trials (SPIRIT); PIP – Parent-Infant Psychotherapy, CAU – care as usual, EAS – Emotional Availability Scales, SSP – Strange Situation Procedure, AQS – Attachment Q-Sort, DC:0–5 – Diagnostic Classification of Mental Health and Developmental Disorders of Infancy and Early Childhood (Interview), CBCL – Child Behaviour Checklist, SFS – Crying Feeding Sleeping Questionnaire, BSCL – Brief Symptom Checklist, ASQ-15 – Anxiety Screening Questionnaire, IES-27 – Scale for Impulsive Behaviour and Emotional Dysregulation of Borderline Personality Disorder, EPDS – Edinburgh Postnatal Depression Scale, M.I.N.I. – Mini International Neuropsychiatric Interview, AAI – Adult Attachment Interview, ET 6–6 R – German developmental test, Bayley-III – Bayley Scales of Infant Development, PSI – Parenting Stress Index, PRFQ – Parental Reflective Functioning Questionnaire

Another questionnaire we have developed also assesses the economic consequences of using health and social care, and requests for early intervention services. Mothers complete the questionnaire at 12 months follow-up. The utilisation documented will be analysed to assess the associated costs (Fig. 3).

### Data collection and monitoring

Researchers responsible for data collection are blind for randomisation and treatment group allocation. All data are stored under pseudonyms until the whole study is terminated. Data on the intervention will be unblinded after the last participant has completed the study. Only pseudonymous data sets will be analysed. There are formal rules for stopping the trial should a severe adverse event (SAE) occur. In the case of SAE the advisory board will be consulted. The consortium leader (LK) will report any SAE to the local ethics committees and all relevant parties. Standard operating procedures (SOPs) will be activated and will lead to exclusion of the participant and potentially to the termination of the trial. The participating scientists and study therapists are trained and qualified in all methods. All study therapists will receive training in PIP according to the SKKIPPI study manual. The SKKIPPI study manual is also the basis for the regular therapy adherence measurements.

The study centres are monitored based on the data collection status (mutual monitoring of the study centres by the study team) to ensure completeness and compliance with ethical considerations.

### Sample size considerations

A power analysis conducted a priori using G*Power was based on the results of a meta-analysis that reported a moderate to large effect size of Cohen’s *d* = 0.64 [16] for between-group differences in maternal sensitivity. Assuming a two-sided α of 0.05 and the computation of an analysis of covariance (ANCOVA) model with maternal sensitivity at 6 weeks as the dependent variable, and maternal sensitivity at baseline as a covariate, this resulted in a required sample size of *n* = 64 (per group) with a power of 90%. Assuming a drop-out rate of 20% [14], the sample size calculates to *n* = 80 per group (total sample size *N* = 160 mother-child dyads). This sample size will also enable the detection of interaction effects considering a moderate moderator effect in the ANCOVA (corresponding to an effect size of *d* = 0.44) with a power of 80%. We initially planned to recruit a sample of 180 infants with a diagnosis of a regulatory disorder and their mothers. However, difficulties in recruitment and funding period constraints meant the planned sample size was reduced to *N* = 160 in June 2020.

### Data analyses

#### General considerations and socio-demographic data

Socio-demographic and clinical information are described at baseline using frequencies and percentages (e.g. categorical variables) or means and standard deviations (e.g. continuous variables), and treatment conditions compared to assess potential baseline differences. The statistical analyses are based on all mother-child dyads included in the study after randomisation (Fig. 1), according to ITT (Intention to Treat) principles. The data to be used in all analyses (unless otherwise stated) is defined as the Full Analysis Set (FAS) and includes all mother-child dyads after randomisation, irrespective of the treatment received. Data from mother-child dyads who drop out of the study are included in the FAS up to the date they withdraw. The Per Protocol (PP) population is a subset of the FAS and includes mother-child dyads who conform to the study plan (i.e. sufficient treatment adherence, no major protocol violations). The safety population is defined as mother-child dyads who have participated in at least one intervention session, and is analysed according to the treatment they received.

#### Primary endpoint

The primary analysis of the primary endpoint (maternal sensitivity measured using the EAS subscale after 6 weeks) will be analysed using ANCOVA based on the FAS population using PIP versus CAU as the factor treatment group, EAS sensitivity at baseline as covariate, and the stratification variable treatment setting. Results will be displayed as adjusted means of the treatment group together with 95% confidence intervals and the respective *p* value (two-sided). The significance level is set at α =0.05. All further analysis is considered exploratory.

A secondary analysis of the primary endpoint will also be carried out using the ANCOVA model based on the PP population. Additional moderator analyses of the efficacy of PIP for a change in maternal sensitivity (EAS) will be conducted with treatment setting as the moderator, i.e. by additionally adding a ‘treatment group’ * ‘treatment setting’ interaction term to the model. Further moderator analyses regarding the efficacy of PIP will comprise maternal attachment type (AAI attachment) or maternal reflective functioning (AAI RF) as moderators.

In addition, models incorporating multiple imputation methods based on the PP population and a hierarchical regression model (including the therapist as a grouping factor and therapist’s adherence as covariate) will be used for sensitivity analyses of the primary outcome. To investigate the longitudinal change of the primary endpoint, linear mixed models (LMMs) incorporating both measurement points for each outcome (FAS population) will be computed. To explore further effects of additional predictors (such as the therapists carrying out the interventions), the models will be compared based on model fit values (AIC, the Akaike Information Criterion), with these predictors included in the model term.

#### Secondary endpoints

Primary analyses of the secondary endpoints replicate the computation of the models for the primary endpoint, as either ANCOVAs or logistic regressions (depending on the continuous or dichotomous scale of the respective endpoint). Group differences in the children’s attachment organisation (SSP/ AQS, binarised evaluation: secure/ insecure attachment), as well as all responder analyses (EAS sensitivity, EPDS, PRFQ, PSI at T1 and T2; SFS at T1, CBCL at T1 and T2), will be computed as logistic regressions adjusted for the stratification variable treatment setting with presentation of odds ratios. Should no cut-off values be available, dichotomous response variables for these measures will be computed using the Reliable Change Index (RCI; RCI > 1.96) [45]. Linear mixed models will be used to analyse the change in the secondary endpoints (maternal stress level, reflective functioning (PRFQ), and infantile symptoms) during the course of the study (12-months follow-up) across all measurement points.

Additional sensitivity analyses will be performed for health economic parameters as secondary endpoints, varying unit-cost assumptions for documented health care utilisation within realistic ranges. All secondary analyses of the primary and secondary endpoints are considered exploratory.

#### Missing data

After written informed consent and randomisation, participants can be excluded from the analyses only if there has not been any intervention, no data have been collected after randomisation, or at the participants’ express request after withdrawing from the study during the data collection phase. Primary statistical analyses of the primary and secondary endpoints are based on the FAS population without the imputation of missing data. For a sensitivity analysis, missing values for the primary endpoint will be imputed using Multiple Imputation methods for linear regression based on Markov chain Monte Carlo (MCMC) simulations. Imputation variables will be selected blinded for the intervention groups, and at least 5000 iterations and 100 imputations will be calculated.

## Discussion

The present RCT investigates the efficacy of a manualised focus-based short-term psychotherapeutic intervention that we have developed, and the efficacy of dyadic psychotherapeutic treatment at home. This study’s main strength is the longitudinal investigation of PIP in different settings. By focusing on affected mother-child dyads in specific dyadic mentalisation-based treatment, the study makes a significant contribution to society and health services. As part of the present RCT, a broad education about the occurrence of regulatory disorders and postpartum mental disorders, and their psychotherapeutic treatment options will be provided to the general public. With its focus on the treatment of mother and child, the study has the potential to improve the future care situation for affected dyads and their families. Treatment in the home environment may also increase the families’ well-being, preventing malignant transmissions of attachment and interaction patterns.

A further benefit lies in the psychotherapeutic intervention itself, since PIP aims to reduce psychological symptoms and improve the attachment between mothers and infants, and will promote child development that is positive in the long term [15]. We expect that the children will develop more resilience and healthier strategies (than without this intervention), and have less probability of developing (mental) illness during childhood. At the same time, improved psychosocial well-being and a higher level of life satisfaction are expected to accompany the reduction of symptoms through the PIP intervention (but also through routine intervention) [1]. PIP interventions are designed as short-term interventions, and the accompanying health economic analyses will examine their possible efficiency in the German health care system for the first time. Compared with a new study by Georg et al. [12], where PIP is administered four times in a 12-week period, this study has the advantage of short-term therapy with 12 sessions over a period of 6 weeks. In addition, the primary outcome of this study is an assessment of maternal sensitivity. This interaction variable is one of the key elements for the healthy development of infants [8–10, 22] and could help us to make stronger predictions about the efficacy of PIP than focusing on symptom reduction [12].

One issue that has not yet been discussed is the transgenerational transmission of malignant patterns. PIP focuses on the mother-infant interaction, which is partly influenced by the mother’s past. Developing a reflective and resourceful type of interaction, as well as reducing children’s symptoms, may also strengthen the mother’s inner resources. Reflection and mentalisation could protect the infant from past maternal trauma [6]. Another advantage of this study is its use of a novel diagnostic classification system for children’s symptoms. The DC: 0–5 [5] and the parent interview we have developed allows differential diagnosis in early childhood. The standard diagnostic classification systems (e.g. ICD-10 and DSM-V) do not cover infancy thoroughly and sufficiently and can only be used for adults, teenagers and older children.

### Potential limitations

One disadvantage is that the study focuses only on the psychiatric care of mothers and their children, and does not include treatment options for fathers. No statement can therefore be made about paternal PIP efficacy (compared to routine therapy). In addition, the father’s involvement and processing throughout the therapeutic process, and the respective outcomes, cannot be evaluated. All participants come to the study centres of their own accord, usually with a wish for treatment. This study cannot make assumptions about dyads sent to the study centres, for example families referred by Child Protective Services. Their motivation for receiving treatment might differ from mothers who seek out help themselves. Another limitation concerns the health economic analysis: most of the expected beneficial long-term consequences will not be covered by the study period. As a result, it is likely that the intervention will not be considered cost-effective without taking into account assumptions about possible long-term effects. To avoid this, a much longer follow-up period would be needed, covering the whole childhood and beyond. Apart from cost-effectiveness, it would be desirable to evaluate the long-term effects of PIP. Unfortunately, this will not be realisable within the SKKIPPI project.

### Implications

This study will provide evidence about the efficacy of PIP for infants with regulatory disorders, which addresses the lack of good-quality randomised controlled trials investigating the efficacy of psychotherapy for mothers and their babies with regulatory disorders in Germany. Caregivers and their infants can only be seen as a system and therefore, only be treated together to implement positive interactions, enhancing sensitivity and attachment security. Moreover, lowering the infant’s risk of developing chronic mental health problems is one of the main issues in the present RCT. This study is the first approach to investigate the efficacy of PIP for inpatient and outpatient psychiatric treatments and home treatment for infants and their mothers and, hopefully, to show novel paths for health care.

### Trial status

The recruitment for the trial started in March 2019 and is expected to be completed in April 2022. This protocol is version 1.0 and dated 8th January 2021.

## Data Availability

Not applicable.

## Abbreviations

AAI: Adult Attachment Interview
ANCOVA: Analysis of covariances
ASQ: Anxiety Screening Questionnaire
Bayley-III: Bayley Scales of Infant Development, Third Edition
BSCL: Brief Symptom Checklist
CAU: Care as usual
CBCL: Child Behaviour Checklist
DC: 0–5: Diagnostic Classification of Mental Health and Developmental Disorders of Infancy and Early Childhood
EAS: Emotional Availability Scale
EPDS: Edinburgh Postnatal Depression Scale
ET6–6-R: German development test
FAS: Full Analysis Set
IES-27: Scale for Impulsive Behaviour and Emotional Dysregulation of Borderline Personality Disorder
ITT: Intention-to-treat
M.I.N.I.: Mini International Neuropsychiatric Interview
PIP: Parent-Infant Psychotherapy
PRFQ: Parental Reflective Functioning Questionnaire
PROBE design: Prospective randomised, open blinded endpoint
PSI: Parenting Stress Index
RCT: Randomised controlled intervention trial
SEGC: Institute for Social Medicine, Epidemiology and Health Economics of the Charité
SFS: German questionnaire Crying, Feeding, Sleeping [Schreien, Füttern, Schlafen]
SKKIPPI: Evaluation of Parent-Infant Psychotherapy: epidemiologic cohort study and intervention studies
SSP: Strange Situation Procedure
